# Race and Final-600 m Speed Response to Distance and Track Condition in Thoroughbred Flat Racing

**DOI:** 10.3390/ani16101433

**Published:** 2026-05-08

**Authors:** Kylie A. Legg, Michaela J. Gibson, Chris W. Rogers

**Affiliations:** School of Veterinary Science, Massey University, Private Bag 11 222, Palmerston North 4410, New Zealand; m.gibson@massey.ac.nz (M.J.G.); c.w.rogers@massey.ac.nz (C.W.R.)

**Keywords:** horse, musculoskeletal injury, modelling, turf track condition

## Abstract

The temporal and spatial distribution of speed varies with race distance and track condition, influencing where mechanical demand peaks within a race. This study aimed to describe variation in race-day speed with race distance and track condition. Race-day data from 18 flat racing seasons on turf and synthetic surfaces (2007/8–2024/25, *n* = 200,601 starts in 3389 races) were used to examine initial speed (from barriers until the final 600 m), final-600 m speed, race distance (m), turf track condition score (TCS, 1–10), horse rating and carried weight (kg) using a general linear model. The pattern of racing was consistent for races ≥ 1000 m. Final-600 m speed at all distances was 0.41 (95% CI 0.41–0.42) m/s faster than initial speed (*p* < 0.001). Both speeds decreased with increasing race distance, but this decrease was smaller for final-600 m speed (β = −0.15 [95% CI −0.16–−0.15] m/s per 200 m) than for initial speed (β = −0.17 [95% CI −0.18–−0.17] m/s per 200 m, *p* < 0.001). Speeds had a negative quadratic relationship with TCS (differing in magnitude and intercept, *p* < 0.001), with a rapid decline at TCS 9. Higher horse rating increased speed, while greater carried weight reduced speed (both *p* < 0.001). The higher race final-600 m speeds were less affected by distance, indicating that mechanical demand is higher late in the race and load accumulation is not uniform.

## Simple Summary

Musculoskeletal injuries are the most common injury in Thoroughbred racehorses and occur due to the accumulation of mechanical load during racing and training. Mechanical load increases with higher speed, such that periods of high speeds impose greater mechanical demand on the limb than slower speed work. Limb loading is often assumed to accumulate uniformly across a race; however, changes in speed within a race may alter where peak loading occurs. Data from 200,601 race starts were analysed to assess how average speed changed during races of different distances and track conditions. Horses consistently ran faster in the final 600 m of all races than earlier in the race. Longer races resulted in slower speeds, but the late-race speed was less affected by race distance. Both early- and late-race speeds declined on heavier turf track conditions, particularly on very wet tracks. Higher rated horses ran faster, while increasing the weight carried reduced speed. These findings indicate that limb loading peaks late in the race rather than accumulating uniformly, highlighting a period of elevated mechanical demand that may be relevant to injury risk.

## 1. Introduction

Musculoskeletal injury (MSI) is the most common reason for involuntary loss of horses in the Thoroughbred racing industry, with catastrophic fracture accounting for most racing related fatalities. Both MSI and fatality pose substantial welfare risks to both horses and jockeys and results in direct and indirect economic losses including compromised social licence to operate [1,2]. Most MSI, including catastrophic fractures, are associated with repetitive bone loading and the accumulation of microdamage, termed the ‘fatigue life’ of bone [3,4,5]. Therefore, minimising MSI and identifying ‘at risk’ horses is a major focus for the Thoroughbred industry and has become an emerging research area [6,7].

Epidemiological studies consistently support the role of cumulative bone loading and fatigue in MSI risk. Older horses, longer race distances, firmer track surfaces and higher rated races are associated with higher risk of fatal MSI [8,9]. Longer race distances present a greater ‘time at risk’ and higher accumulation of load cycles. Firmer tracks further elevate MSI risk due to the lack of cushioning and yield increasing the magnitude of loads on the limb [8,10,11]. Race speed is a known risk factor for MSI via increased peak bone strain and is positively associated with both firmer track surfaces and higher rated races [6,9,11,12,13,14].

Speed and stride-level variables can be used to model the accumulation of fatigue life during training and racing [6,7]. However, when these variables are reduced to average race or final sectional measures, it can be assumed that load accumulates uniformly and scales linearly with MSI risk. These assumptions obscure the observed temporal and spatial variability of speed within a race, influencing the mechanical load on the limb. Speeds fluctuate according to race tactics as well as with race distance and track condition [11]. Track geometry and curvature also alter limb loading patterns due to increased mediolateral and torsional loads as the horse rounds the bend [15,16]. In New Zealand, the majority of fatal race-day MSI events occur around the final turn or on the home straight [17,18], suggesting that the effective limb load increases at these locations in the race. Therefore, fatigue accumulation may remain sub-critical for the majority of a race and only rise steeply once critical loads are exceeded.

This concept of a limiting stress is well established in materials science and biomechanics. In theory, cyclic loads below a certain threshold contribute minimally to fatigue damage, whereas loads above this limit accelerate microdamage formation and markedly reduce fatigue life [19,20]. Bone exhibits a similar behaviour where repeated high-strain or high strain rate cycles disproportionately drive microdamage accumulation, with fatigue life decreasing exponentially with increasing load [21]. Thus, short periods of very high speed or limb loading may be responsible for a disproportionate share of fatigue accumulation, even within the high speeds of competitive races. Given this nonlinear loading behaviour, understanding how speed varies within a race is central to understanding limb loading patterns and interpreting MSI risk. Therefore, the aim of this study was to describe variation in race-day speed with race distance and track condition.

## 2. Materials and Methods

Race-day data were provided by New Zealand Thoroughbred Racing (NZTR), the governing body for Thoroughbred racing in New Zealand. These data included every race start conducted at Thoroughbred racetracks across New Zealand between 7 February 2007 and 30 July 2025 (18.5 racing seasons). Every horse start included detailed track and race specific details including racetrack location, race type (flat or jumps), track surface type (turf or synthetic), track condition score (TCS), penetrometer reading, descriptive track conditions, race distance (m) and individual race and final-600 m sectional times (s).

Racetracks in New Zealand are predominately turf, with only three recently developed (since 2019) synthetic tracks. Racetrack configuration in New Zealand is fairly consistent in terms of geometry and topography, with soil type and environmental factors likely to provide the greatest differences between turf track locations [22]. Turf track condition scores are based on the average of quantitative penetrometer measurements taken every 200 m around each track on the morning of every race day. Penetrometer measurements provide repeatable measured of racetrack firmness which are converted to a TCS, with categories from 1 (fast)–10 (heavy) as described in Legg, Gibson, Gee and Rogers [11].

Horse-specific data including horse rating (domestic rating), carried weight (kg) and finishing position of the horse were also included. Horse rating is a dynamic measure of performance where each horse is assigned a numerical rating that represents their assessed racing ability and is recalculated within 2 days of a horse’s most recent race start [23]. This allows horses to be ‘grouped’ into similar classes and handicap weights to be allocated with higher rated horses having greater racing success. Only flat races were used for analysis in this study.

Data were tested for normality using the Kolmogorov–Smirnov test for normality (*p* < 0.05), which is appropriate for assessing distributional assumptions in large datasets used for linear regression. Descriptive statistics were used to summarise, describe and screen the data with differences between variables tested with the Kruskal–Wallis test for non-parametric data.

Mean race speeds were calculated from each horse’s official race time and total race distance. Mean final-600 m speeds were calculated from the recorded individual time to cover the final 600 m sectional. Mean initial speed was calculated as the speed for the race segment prior to the final-600 m (from the barriers until the final-600 m of the race). A category “race phase” was created to classify each observation as either the mean whole race speed, mean initial speed or mean final-600 m speed for each horse. Speeds < 12 m/s and >22 m/s were excluded as biologically implausible or indicative of abnormal race performance (e.g., pulling up in-race).

Race distances were grouped into 200 m intervals to enable comparison of mean initial and mean final-600 m speeds between races of different lengths. Within each bin, summary statistics (mean and SD) of race distances and both speed types were calculated.

A general linear model was used to assess the relationship between speed and the fixed effects of race phase (mean initial or mean final-600 m speed), TCS, race distance, carried weight and horse rating. Track condition score was modelled as a quadratic numeric variable within this model [11], so only race starts on a turf surface were used for the multivariable model. Univariable analysis was used to screen the relevant predictor variables at *p* ≤ 0.2. Variables which improved model fit (*p* < 0.05) were selected using a forwards and backwards selection procedure in the multivariable model. The random effect of horse in the model was tested for, but due to the large number of observations, the random effect was insignificant and removed from the final model. A full model including TCS, TCS^2^ and all two-way interactions between race phase, race distance and TCS^2^ were compared to a reduced model which retained TCS^2^ only as a main effect, and with a model which tested all TCS^2^ interactions. Information criteria were lower for the full model in all tests (ΔAIC = 2527.3; ΔBIC = 2494.7 and ΔAIC = 612.5; ΔBIC = 601.7), so the full model was retained. A likelihood-ratio test also supported the full model (χ^2^ = 197.4, *p* < 0.001). A three-way interaction between the variables of interest did not improve model fit.

All statistical analyses were conducted using R Build 403 (2026.01.1, R Core Team, 2025) with the level of significance set at *p* < 0.05.

## 3. Results

There were 200,601 starts in 27,197 flat races on 3389 race days in New Zealand which had race and final-600 m times recorded between 7 February 2007–30 July 2025. These races involved 20,695 horses on 58 different racetracks, with race starts evenly distributed between sexes (*n* = 102,499 male starts, 51%). Over this period, most starts were on turf (95%) on a median TCS of 5 (IQR 4–8), with the remainder raced on a synthetic surface (*n* = 9084, 5%). The median race distance was 1400 m (IQR 1200–1600 m) for both turf and synthetic surfaces. The median domestic rating for turf was 60 (IQR 50–67) and for synthetic was 55 (IQR 48–62). The median carried weight for both surfaces was 56.5 kg (IQR 55.0–58.0).

Mean whole race speed for all distances and surfaces was a median of 16.1 m/s (IQR 15.6–16.5 m/s). Mean initial speed was slower at 16.0 (IQR 15.5–16.4) m/s and mean final-600 m speed faster at 16.4 (IQR 15.6–17.0) m/s than whole race speed (*p* < 0.001). Horses raced faster on synthetic surfaces than turf, with mean whole race and mean final-600 m speeds comparable with a TCS of 1–2 (Table 1).

**Figure 1:**
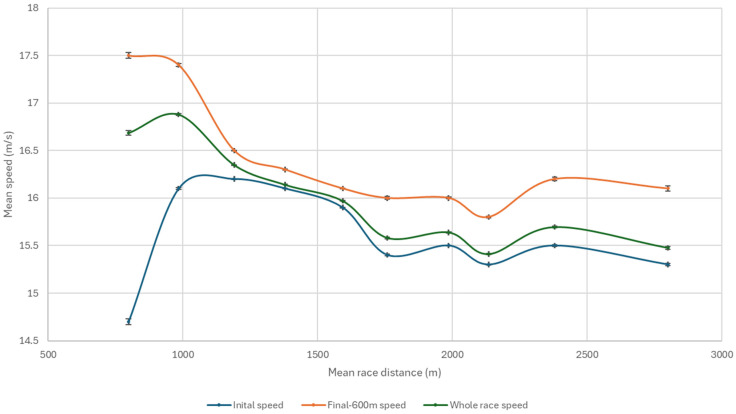
Mean speed (±SE) for the whole race, and initial phase (barriers until the final-600 m) and final-600 m sectional of races for mean race distances categorised into 200 m intervals. Means represent the central value for each 200 m interval and error bars represent the standard error of speed for that interval. Lines connecting the points are included for visualisation only.

Mean (±SD) speed peaked at 17.5 ± 0.8 m/s during the final-600 m sectional in sprint races (≤1000 m) (Figure 1). Mean speed for the initial 200–400 m (in races ≤ 1000 m) was 16.0 ± 0.8 m/s, before settling at 16.1 ± 0.6 m/s for races between 1001 and 1600 m and was slower at 15.4 ± 0.6 m/s for longer races (1601–3200 m, *p* < 0.001). Mean speeds at all distances were greater in the final-600 m sectional than either whole race speed, or mean initial speed (Figure 1, *p* < 0.001).

Univariable analysis confirmed that all relevant effects (race phase, race distance, TCS, domestic rating and carried weight) significantly affected the speed of the horse (Supplementary Table S1). There were significant interactions between race phase (initial or final-600 m speed) and both race distance and TCS (*p* < 0.001) which were retained in the final multivariable model (Table 2).

**Figure 2:**
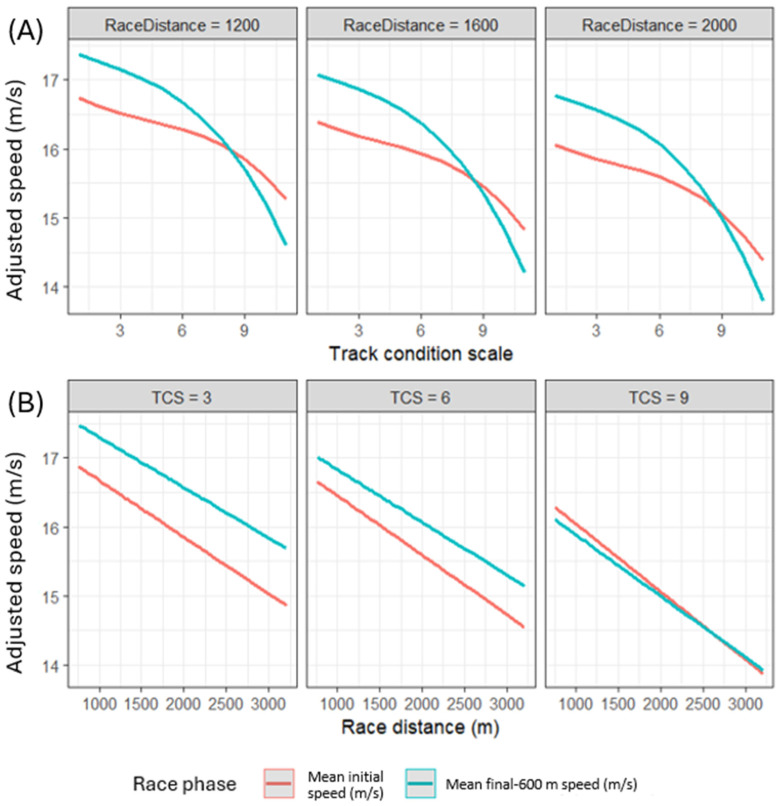
Mean initial and mean final-600 m speed (m/s) of Thoroughbred horses in flat races (n = 190,817) with respect to (**A**) track condition score (TCS) and (**B**) race distance (m) in a multivariable general linear regression model with fixed effects of domestic rating and carried weight (linear). Values represent model-adjusted mean speeds estimated from the model with all other variables held constant at their mean or reference values.

Multivariable analysis confirmed that mean final-600 m speed was higher than mean initial speed for all races (Table 2). Speed was negatively associated with longer race distances, with mean final-600 m speeds less affected by longer race distances than mean initial speeds. Speed had a curvilinear relationship with TCS, indicating that speed decreased with increasing TCS, but the rate of decrease increased at higher TCS values (Figure 2). Final-600 m speeds were more affected by higher TCS than initial speeds. Both the slope and curvature of the speed–TCS relationship changed with race distances and between race phase (Figure 2). Higher domestic rated horses had higher speeds, and greater carried weights were associated with lower speeds.

## 4. Discussion

The analysis of race, initial and final-600 m speeds revealed that the pattern of racing in New Zealand was similar across track surfaces (turf and synthetic) and race distances. Mean speeds suggested that initially horses jumped out of the starting gates, then established relative positions and maintained moderate speeds until increasing speed in the final 600 m, similar to the pattern described for racing in Japan [24]. This pattern remained consistent up to a turf track TCS 9, when tracks were classified as “heavy”. At this TCS, it appeared that fatigue played a greater role in mean final-600 m speeds, with the speed becoming slower than the initial sections of the race. Even though horses in longer-distance races started at slower speeds, this inflexion point relative to TCS remained relatively consistent across race distances. Horse level performance indicators behaved as expected, with higher rated horses associated with faster speeds and greater carried weights associated with slower speeds, reflecting handicapping practices that attempt to neutralise natural performance advantages.

Across jurisdictions, there is variation in whether race distance is a risk factor for MSI or catastrophic MSI [9,12,25] and generally a lack of precision in quantifying “going” on turf surfaces with only a few jurisdictions using quantitative rather than qualitative scales [11,26]. Synthetic tracks in this study were comparable to a turf TCS 1–2; however, differences in surface types (turf, synthetic, dirt) and their preparation may result in both a varied speed response and differences in the hoof–surface interaction, thus altering the effective limb load. Turf TCS exerted a curvilinear effect on speed and interacted with both distance and race phase, suggesting that softer tracks may moderate speed but do not uniformly reduce late-race loading (final-600 m speed). These insights help reconcile jurisdictional differences in the reported risk of race distance on MSI [9] and underline that risk attribution depends on local racing characteristics and final-600 m speed, not distance alone. Additionally, use of final-600 m speed to model whole race load may overestimate the total load over a whole race.

Mean race speed on turf surfaces decreased with increasing distance and higher (softer) TCS, consistent with previous studies reporting race speed [6,11]. However, the mean final-600 m speed was less variable than mean initial speed, consistent with horses reaching near-maximal effort at the end of the race [24]. On New Zealand tracks, the final-600 m occurs as the horses round the final turn and enter the home straight. Track curvature has been previously associated with increases in speed, with concurrent decreases in stride length and increased stride frequency, which, combined with the additional centrifugal force of the horse negotiating the corner, affects the loading of the limb [16,27]. This profile aligns with a threshold-loading pattern, where most of the race may occur near or below limiting stress, with higher loads concentrated around the last bend and home straight, sections where the majority of catastrophic MSI in New Zealand have been recorded [17,18].

Greater relative speeds were achieved on synthetic surfaces than on turf surfaces with comparable race distances, carried weights and domestic ratings. Within the literature, there are limited data comparing racing speeds between synthetic and turf surfaces, but under training conditions, the same trend has been observed [28]. Surface type and condition can influence not just speed, but also horse stride variables and movement patterns, with reported changes in stride length of 0.18 m due to different track surface preparations and ‘firmness’ on dirt tracks [29]. The final-600 m speeds on synthetic tracks were equivalent to speeds achieved on turf tracks with TCS 1–2, which have been documented as a higher risk category for MSI and catastrophic MSI [8,9]. However, it appears that horses racing on synthetic surfaces in New Zealand and internationally do not necessarily have a higher risk of MSI, despite the higher speeds observed [9,13]. This could be due to altered limb loading mechanics observed in synthetic surfaces compared to turf, which may reduce injury risk [30]. Jurisdictional differences in whether synthetic tracks confer higher, lower or similar MSI risk [9,25,31,32] highlight that surface effects are not uniform, and limb loading is influenced by the pattern of racing and difference in track surface, geometry and track preparation within each jurisdiction [2,30].

Shorter distance races, 800 m and 1000 m, are generally restricted to 2-year-old racing, with the shorter 800 m races offered at the start of the season. In the univariable data, these races were characterised by higher mean final-600 m speeds, but slower mean initial speeds. This pattern reflects the inexperience of the 2-year-olds when jumping out of the starting gates, as dramatically shown with the 800 m race distance, rather than a distance effect per se. The classic 1200 m sprint distance demonstrated higher speeds and less differentiation between mean initial and mean final-600 m speed. Interpretation of these results requires caution as the initial speeds of these races were calculated from shorter opening sectionals (200 m in 800 m races, 400 m in 1000 m races) than the longer-distance races, inflating the apparent contrast between early- and late-race speed.

The multivariable data clearly demonstrated greater mean race, initial and final-600 m speed with the shorter sprint distances. It has been suggested that the higher speeds in sprinters are associated with a higher stride frequency [33], which would increase both the quantity and magnitude of limb loading cycles, potentially elevating MSI risk and accumulation of fatigue life. In some racing jurisdictions, sprint distances were associated with increased risk of race-day catastrophic MSI, though for other jurisdictions there was no race distance effect [9]. Sprint races in many jurisdictions tend to occur in the summer months when TCS is lower, and the surface is harder, exacerbating limb loading [34]. Therefore, differences in surface properties (such as cushioning) which alter the hoof–surface interaction to help dissipate loading at higher speeds become more important. On dirt tracks, hardness and moisture content also affect both stride frequency and stride length, which adds to the difficulties in assessing the effect of a simple number of cumulative load cycles [29]. These data indicate that it may be not only be loads above a certain race speed or race distance that has an impact on describing risk, but a complex interaction of speed, surface properties and track geometry.

Horses in races > 1000 m settled into a steadier, slower pace for the majority of the race. This may provide lower relative limb loading in these races despite the greater overall time on track. In exercise physiology, the concept of training impulses (TRIMPs) has been used to demonstrate this effect, with time above a certain physiological threshold being more important than the cumulative duration of the competition event [35]. This approach could also be applied to load in the distal limb during racing, with only the cumulative time above the minimal threshold, or in engineering terms “limiting stress”, contributing to the accumulation of fatigue life or effective time “at risk” [19,20,21].

The longer-distance races still involved a pronounced late-race speed. Although the mean final-600 m speed was not as fast as that observed in the shorter sprints, it appears to represent the highest-load phase of the race for these events. Together, these patterns suggest that sprint races concentrate loading into a short, high-intensity window, whereas longer-distance races combine prolonged moderate loading with a final high-load phase, producing distinct but convergent pathways of mechanical demand that may help explain variation in MSI risk across race types [9].

Within this framework, differences in stride mechanics provide an additional layer of complexity when considering cumulative limb loading during racing. Speed can be increased through changes in stride frequency, stride length, or a combination of both. However, these variables are not independent, and the strategy used to attain higher speed has implications for both the magnitude of load per stride and the number of loading cycles accumulated over a given distance. Horses achieving higher speeds primarily through increased stride length may not experience a proportional increase in load cycle count, highlighting that faster racing does not necessarily equate to a greater number of loading cycles. This distinction is critical when interpreting cumulative load, as microdamage accumulation is dependent on both the magnitude and the number of load cycles. Consequently, two races covering identical distances may impose markedly different mechanical demands depending on how speed is distributed across strides and race phases. These differences are further exacerbated by the interaction with track surface properties (both type and ‘firmness’) and geometry, which have both been shown to influence not only race speed, but stride length and frequency.

Future surveillance and prevention of MSI programmes could prioritise late-race sectional (last bend/straight) where speeds are both highest and least variable. Many race trainers recognise that sprints may concentrate rather than prolong high loads, while staying races may include mid-race relief that permits quick back-up between races. Future work could investigate the link between final 600 m speeds with in-race MSI location data (e.g., last bend vs. finish line) and test load-threshold models. Given the nonlinear loading behaviour of the equine limb, understanding where and when horses reach high speeds within a race is essential to interpreting MSI risk and building models that reliably predict limb load.

This analysis provides large scale data describing variations in race speed within a race and between race distances. However, due to the nature of the data, only whole race and final 600 m sectional times were available for analysis, restricting the ability to characterise speeds instantaneously throughout an entire race. Future research incorporating 200 m sectional or GPS-based data would enable a more detailed analysis. Track condition and surface type were reliably and quantitatively recorded for the 18 years of the study; however, specific course geometry and more detailed variations within a track surface could not be quantified. Although limb loading and its relationship with injury risk are discussed conceptually, no direct biomechanical measurements were collected in this study. As such, inferences regarding limb-level mechanical loading are based on established relationships between speed, race dynamics, and musculoskeletal load rather than direct kinetic or kinematic assessments.

## 5. Conclusions

Mean final-600 m sectional speed was higher in all races on both turf and synthetic surfaces. On turf surfaces, race speed decreased as distance increased; however, the mean final-600 m speed had a smaller distance effect, consistent with horses accelerating to near-maximal effort late in the race. These data indicate that the use of total race speed and race distance may create bias within risk factor models for race-day MSI and catastrophic MSI, as the majority of the effective loads during a race may occur during the final 600 m. Within risk factor models for race-day injury, final 600 m speed in association with track surface type, TCS and race distance as descriptors of the effect of fatigue may provide a more elegant reflection of limb load and loading.

## Figures and Tables

**Table 1:** Median (IQR) speed at different race phases (mean whole race, mean initial and mean final-600 m) of horses racing on different surfaces and track condition scores in New Zealand flat racing between 7 February 2007–30 July 2025.

Race Surface	*n* (Starts)	Median (IQR) Race Phase Speed (m/s)					
		Whole Race		Initial		Final-600 m	
Synthetic	9084	16.6	(16.3–16.9) ^a^	16.3	(16.0–16.6) ^a^	17.0	(16.6–17.4) ^a^
Turf (all)	191,517	16.1	(15.6–16.5)	15.9	(15.5–16.3)	16.4	(15.6–16.9)
TCS 1	8	16.9	(16.5–17.2) ^ab^	16.7	(16.5–17.0) ^b^	17.2	(16.7–17.4) ^abc^
TCS 2	1252	16.5	(16.2–16.8) ^bc^	16.3	(15.9–16.6) ^c^	17.0	(16.7–17.4) ^a^
TCS 3	25,840	16.5	(16.2–16.8) ^c^	16.3	(15.9–16.6) ^c^	17.0	(16.5–17.3) ^b^
TCS 4	46,500	16.4	(16.1–16.7) ^d^	16.2	(15.8–16.5) ^d^	16.8	(16.4–17.2) ^c^
TCS 5	25,383	16.3	(16.0–16.6) ^e^	16.1	(15.8–16.4) ^e^	16.7	(16.2–17.1) ^d^
TCS 6	15,126	16.2	(15.9–16.4) ^f^	16.0	(15.7–16.3) ^f^	16.4	(16.0–16.8) ^e^
TCS 7	17,054	16.0	(15.6–16.3) ^g^	15.9	(15.5–16.2) ^g^	16.2	(15.7–16.6) ^f^
TCS 8	13,484	15.8	(15.5–16.1) ^h^	15.7	(15.4–16.1) ^h^	15.9	(15.4–16.3) ^g^
TCS 9	9194	15.6	(15.2–15.9) ^i^	15.6	(15.2–16.0) ^i^	15.6	(15.1–16.1) ^h^
TCS 10	36,976	15.1	(14.7–15.5) ^j^	15.3	(14.8–15.7) ^j^	14.9	(14.3–15.5) ^i^

^a,b,c,d,e,f,g,h,i,j^ superscripts denote differences between speeds (*p* < 0.05).

**Table 2:** Multivariable general linear regression model of the association of speed (m/s) with predictor variables: race phase (initial or final-600 m), track condition score (TCS + TCS^2^), race distance (m), domestic rating of the horse and carried weight (kg). Model estimates represent adjusted regression coefficients indicating the expected change in speed associated with each predictor, conditional on all other terms in the model, with non-focal variables held at their reference or mean values. Model includes interactions between race phase with race distance and TCS^2^, and race distance with TCS^2^. Base model uses mean initial speed as reference.

Predictors	Speed				
	Estimates	CI	Std. Error	t Value	*p*
(Intercept)	17.8	17.7–17.9	0.045	394.8	<0.001
Race phase (final-600 m speed)	0.48	0.45–0.51	0.017	28.2	<0.001
Race distance (per 200 m)	−0.17	−0.18–−0.17	2.2 × 10^−5^	−40.6	<0.001
TCS	−0.24	−0.27–−0.21	0.016	−15.0	<0.001
TCS^2^	0.04	0.03–0.04	0.002	17.8	<0.001
Flat rating	0.011	0.011–0.012	7.6 × 10^−5^	150.9	<0.001
Carried Weight (kg)	−0.01	−0.011–−0.009	3.8 × 10^−4^	−25.8	<0.001
Final-600 m speed × Race Distance (per 200 m)	0.019	0.017–0.021	5.2 × 10^−6^	18.2	<0.001
Final-600 m speed × TCS	0.06	0.05–0.07	0.005	12.8	<0.001
Final-600 m speed × TCS^2^	−0.016	−0.017–−0.016	3.8 × 10^−4^	−43.0	<0.001
Race Distance (per 200 m) × TCS	6.20 × 10^−3^	3.4 × 10^−3^–9.1 × 10^−3^	7.3 × 10^−6^	4.3	<0.001
Race Distance (per 200 m) × TCS^2^	−9.50 × 10^−4^	−1.2 × 10^−3^–−7.4 × 10^−4^	5.5 × 10^−7^	−8.7	<0.001
TCS × TCS^2^	−2.20 × 10^−3^	−2.4 × 10^−3^–−2.1 × 10^−3^	9.0 × 10^−5^	−24.8	<0.001
Observations	190,817				
R^2^	0.6				

## Data Availability

Data are available in a public, open access repository. All data used in this study is freely available in a non-collated format on the website of New Zealand Thoroughbred Racing Inc. (www.nzracing.co.nz, accessed on 1 January 2026).

## Supplementary Materials

The following supporting information can be downloaded at: https://www.mdpi.com/article/10.3390/ani16101433/s1, Table S1: Mixed effect univariable linear regression model of the association of speed (m/s) in the final 600 m of a race with predictor variables; track condition score (TCS + TCS^2^), race distance (m) and domestic rating of the horse with the random effect of horse included in the model.

## Abbreviations

The following abbreviations are used in this manuscript:

MSI	Musculoskeletal injury
NZTR	New Zealand Thoroughbred Race
TCS	Track condition score (a scale from 1 to 10, based on penetrometer readings)

## References

[1] Heleski C., Stowe C.J., Fiedler J., Peterson M.L., Brady C., Wickens C., MacLeod J.N. (2020). Thoroughbred racehorse welfare through the lens of ‘social license to operate—With an emphasis on a U.S. perspective. Sustainability.

[2] Legg K.A., Gee E.K., Breheny M., Gibson M.J., Rogers C.W. (2023). A bioeconomic model for the Thoroughbred racing industry—Optimisation of the production cycle with a horse centric welfare perspective. Animals.

[3] Verheyen K.L.P., Wood J.L.N. (2004). Descriptive epidemiology of fractures occurring in British Thoroughbred racehorses in training. Equine Vet. J..

[4] Whitton R., Ayodele B., Hitchens P., Mackie E. (2018). Subchondral bone microdamage accumulation in distal metacarpus of Thoroughbred racehorses. Equine Vet. J..

[5] Martig S., Lee P.V., Anderson G.A., Whitton R.C. (2013). Compressive fatigue life of subchondral bone of the metacarpal condyle in thoroughbred racehorses. Bone.

[6] Wong A.S.M., Morrice-West A.V., Whitton R.C., Hitchens P.L. (2022). Changes in Thoroughbred speed and stride characteristics over successive race starts and their association with musculoskeletal injury. Equine Vet. J..

[7] Morrice-West A.V., Wong A.S.M., Hitchens P.L., Whitton R.C. (2025). The impact of cumulative bone fatigue on musculoskeletal injury risk in racing Thoroughbreds. Vet. J..

[8] Bolwell C.F., Rogers C., Gee E., McIlwraith W. (2017). Epidemiology of Musculoskeletal Injury during Racing on New Zealand Racetracks 2005-2011. Animals.

[9] Hitchens P.L., Morrice-West A.V., Stevenson M.A., Whitton R.C. (2019). Meta-analysis of risk factors for racehorse catastrophic musculoskeletal injury in flat racing. Vet. J..

[10] Parkin T.D.H., Clegg P.D., French N.P., Proudman C.J., Riggs C.M., Singer E.R., Webbon P.M., Morgan K.L. (2004). Race- and course-level risk factors for fatal distal limb fracture in racing Thoroughbreds. Equine Vet. J..

[11] Legg K.A., Gibson M.J., Gee E.K., Rogers C.W. (2025). Turf track surface interaction with speed and musculoskeletal injury risk in Thoroughbred racehorses. Equine Vet. J..

[12] Gibson M.J., Legg K.A., Gee E.K., Rogers C.W. (2023). The reporting of racehorse fatalities in New Zealand Thoroughbred flat racing in the 2011/12–2021/22 seasons. Animals.

[13] Gibson M.J., Legg K.A., Gee E.K., Rogers C.W. (2022). Race-level reporting of incidents using an online system during three seasons (2019/2020–2021/2022) of Thoroughbred flat racing in New Zealand. Animals.

[14] Witte T.H., Hirst C.V., Wilson A.M. (2006). Effect of speed on stride parameters in racehorses at gallop in field conditions. J. Exp. Biol..

[15] Peterson M., Sanderson W., Kussainov N., Hobbs S.J., Miles P., Scollay M.C., Clayton H.M. (2021). Effects of racing surface and turn radius on fatal limb fractures in Thoroughbred racehorses. Sustainability.

[16] Parkes R.S.V., Pfau T., Weller R., Witte T.H. (2020). The effect of curve running on distal limb kinematics in the Thoroughbred racehorse. PLoS ONE.

[17] Gibson M.J., Legg K.A., Rogers C.W. (2024). Internal Report-Equine Mortality Review Panel 2023/2024 Thoroughbred Racing Season.

[18] Gibson M.J., Legg K.A., Martinez-Wherle A., Dittmer K., Rogers C.W. (2025). Internal Report—Equine Mortality Review Panel 2024/2025 Thoroughbred Racing Season.

[19] Herasymchuk O.M. (2023). Theoretical Estimation of the Endurance Limit of Metal Materials by the Characteristics of Their Static Strength and Microstructure Based on the Linear-Elastic Fracture Mechanics. Strength Mater..

[20] Pan Q., Lu L. (2026). Fatigue in metals and alloys. Nat. Mater..

[21] Martig S., Chen W., Lee P.V., Whitton R.C. (2014). Bone fatigue and its implications for injuries in racehorses. Equine Vet. J..

[22] Rogers C.W., Bolwell C.F., Gee E.K., Peterson M.L., McIlwraith C.W. (2014). Profile and surface conditions of New Zealand Thoroughbred racetracks. J. Equine Vet. Sci..

[23] New Zealand Thoroughbred Racing (2019). Handicapping Guide.

[24] Takahashi Y., Pfau T., Tsuruoka F., Yoshida T., Edwards W.B., Mukai K. (2025). Determinants of stride parameters in Thoroughbreds racing in Japan. Am. J. Vet. Res..

[25] Georgopoulos S.P., Parkin T.D.H. (2016). Risk factors associated with fatal injuries in Thoroughbred racehorses competing in flat racing in the United States and Canada. J. Am. Vet. Med. Assoc..

[26] Schmitt P.R., Sanderson W., Rogers J., Barzee T.J., Peterson M. (2024). A Comparison of Devices for Race Day Characterization of North American Turfgrass Thoroughbred Racing Surfaces. Animals.

[27] van den Broek M., Chan Z.Y.S., De Bruyne C., Garcia-Alamo K., Skotarek Loch S., Pfau T. (2025). Association between stride parameters and racetrack curvature for Thoroughbred Chuckwagon horses. Sensors.

[28] Horan K., Coburn J., Kourdache K., Day P., Harborne D., Brinkley L., Carnall H., Hammond L., Peterson M., Millard S. (2021). Influence of speed, ground surface and shoeing condition on hoof breakover duration in galloping Thoroughbred racehorses. Animals.

[29] Pfau T., Bruce O.L., Sawatsky A., Leguillette R., Edwards W.B. (2024). Dirt track surface preparation and associated differences in speed, stride length, and stride frequency in galloping horses. Sensors.

[30] Setterbo J.J., Hubbard M., Garcia T.C., Reese J.L., Morgan J.M., Kim S.Y., Stover S.M., Campbell I.P. (2009). Hoof accelerations and ground reaction forces of Thoroughbred racehorses measured on dirt, synthetic, and turf track surfaces. Am. J. Vet. Res..

[31] Reardon R.J., Boden L., Stirk A.J., Parkin T.D. (2014). Accuracy of distal limb fracture diagnosis at British racecourses 1999-2005. Vet. Rec..

[32] Williams R.B., Harkins L.S., Hammond C.J., Wood J.L.N. (2001). Racehorse injuries, clinical problems and fatalities recorded on British racecourses from flat racing and National Hunt racing during 1996, 1997 and 1998. Equine Vet. J..

[33] Schrurs C., Blott S., Dubois G., Van Erck-Westergren E., Gardner D.S. (2022). Locomotory Profiles in Thoroughbreds: Peak Stride Length and Frequency in Training and Association with Race Outcomes. Animals.

[34] Bruce O.L., Pfau T., Crack L.E., Sawatsky A., Leguillette R., Edwards W.B. (2026). The influence of dirt track hardness on equine limb acceleration and impact attenuation. BMC Vet. Res..

[35] Padilla S., Mujika I., Orbananos J., Santisteban J., Angulo F., Goiriena J.J. (2001). Exercise Intensity and Load During Mass-Start Stage Races in Professional Road Cycling. Med. Sci. Sports Exerc..

