# Supplementary material for: Race and Final-600 m Speed Response to Distance and Track Condition in Thoroughbred Flat Racing
Source: Animals (Basel). 2026 May 8;16(10):1433. doi: 10.3390/ani16101433 (PMC13203147; doi:10.3390/ani16101433)
Supplement: Supplementary file 1 [file animals-16-01433-s001.zip › animals-4290242-supplementary.pdf]

Supplementary Table S1. Univariable general linear regression model of the association of speed (m/s) with predictor variables: race phase (mean initial or mean final-600m speed), track condition score (TCS+TCS^2), race distance (m), domestic rating of the horse and carried weight (kg).

Predictors	**Speed**				
	Estimates	*Std. Error*	*t value*	*p*	*AIC*
Base model	16.1	1.4E-03	11395	<0.001	1047735
Race phase (intercept)	15.9	2.0E-03	8113	<0.001	1034035
Race phase (final-600m)	0.33	2.8E-03	118	<0.001	
Race distance (intercept)	17.3	6.0E-03	2865	<0.001	1008177
Race distance (per 200m)	-0.16	7.7E-04	-204	<0.001	
TCS (intercept)	16.5	9.1E-03	1808	<0.001	803623
TCS	0.10	3.1E-03	33	<0.001	
TCS^2	-0.02	2.3E-04	-104	<0.001	
Flat rating (intercept)	15.5	7.0E-03	2206	<0.001	1041567
Flat rating	0.009	1.1E-04	79	<0.001	
Carried Weight (kg) (intercept)	17.4	0.03	526	<0.001	1046131
Carried Weight (kg)	-0.02	5.9E-04	-40	<0.001	
